# Pre-stimulus Microstates and Bodily Signals Independently Influence Perceptual Awareness at the Discrimination Threshold

**DOI:** 10.1007/s10548-025-01139-6

**Published:** 2025-08-28

**Authors:** Viviana Leupin, Juliane Britz

**Affiliations:** Department of Psychology, University of Fribourg, University of Fribourg, Rue P.-A. Faucigny 2, Fribourg, CH-1700 Switzerland

## Abstract

Perceptual awareness of threshold or multi-stable stimuli varies with the pre-stimulus global state of the brain as indexed by EEG microstates. Similarly, awareness also varies with cyclic fluctuations of visceral signals across the cardiac and the respiratory cycle. It remains to be investigated whether the momentary state of the brain contributes to awareness jointly or independently of the bodily phase. We used an orientation discrimination task to determine to what degree the subjective awareness of a visual threshold stimulus varied with the pre-stimulus microstate, cardiac and respiratory phase and whether the brain and body exerted a joint or independent influence on fluctuations of subjective awareness. We compared the pre-stimulus EEG microstates preceding correct aware and unaware trials for the cardiac and respiratory phase. Our findings indicate that the canonical Microstate D was more prevalent in the unaware compared to the aware condition, and the canonical Microstate A accounted for more variance during inhalation compared to exhalation. The pre-stimulus activation of Microstate D, which is anticorrelated with attentional networks preceded trials in which the stimulus was not perceived. Inhalation was instead associated with Microstate A, suggesting increased arousal during this phase. However, we observed no interaction between the bodily phase and awareness, suggesting that the states of the brain and the body exert independent influence on perceptual awareness at the discrimination threshold.

## Introduction

Stimuli presented at the sensory threshold are equally likely perceived or missed. Since awareness varies randomly from one trial to another independently of the sensory signal, the source of this variability must originate from neural processes that are either a response to the stimulus and/or spontaneous fluctuations in brain activity preceding the presentation of the stimulus. It is crucial to recognize that the brain response to external stimuli is deeply intertwined with its ongoing spontaneous activity, which is not random noise but an informative and structured signal (Arieli et al. 1996).

The pre-stimulus brain state can be used to predict differences in perceptual awareness in humans by employing both local and global indices of EEG activity in experimental paradigms with multi-stable or threshold stimuli. Local measures of EEG frequency power have been widely investigated in relation to perceptual awareness. In particular, power in the alpha rhythm (8–13 Hz) is considered as an index of cortical excitability to which awareness is inversely related, and trial-to trial variations in the pre-stimulus alpha band affect the detection of stimuli at the sensory threshold (Ergenoglu et al. 2004; Hanslmayr et al. 2007; Romei et al. 2008) and their conscious perception irrespective of performance (Benwell et al. 2017) or visual sensitivity (Benwell et al. 2022).

EEG microstates are brief periods (60–120 ms) of stable scalp electrical field configurations (Lehmann et al. 1987); they are a global and hence reference-free EEG measure. EEG microstates at rest are correlated with large-scale resting state networks observed in fMRI (Britz et al. 2010; Van De Ville et al. 2010). This association indicates that periods of stable scalp topography reflect the transitory activity of specific neurocognitive networks operating at the sub-second temporal scale.

In the context of perception, the global measure of EEG microstates provides a complementary approach to local EEG measures. Because the scalp topography remains stable and encompasses all concurrently active intracranial sources, it is possible to identify a single dominant topography which precedes the onset of the stimulus. The momentary state of the brain reflected by the pre-stimulus EEG microstates can determine the fate of upcoming stimuli (Britz et al. 2009, 2011, 2014; Mohr et al. 2005). Studies with ambiguous figures (Britz et al. 2009) and binocular rivalry (Britz and Pitts 2011) show that perceptual reversals vary with the pre-stimulus microstate immediately before the stimulus. The pre-stimulus state of the brain similarly varies with the perceptual outcome of a stimulus presented at the sensory threshold (Britz et al. 2014): pre-stimulus EEG microstates doubly dissociated trials in which the stimulus was correctly identified with and without awareness (Britz et al. 2014). Overall, both local and global spontaneous fluctuations in pre-stimulus brain activity contribute to the perceptual outcome of a stimulus (Britz et al. 2014; Britz and Michel 2011; Ergenoglu et al. 2004; He 2013; Iemi et al. 2019).

The brain continuously adapts its state to both internal and external demands, and bodily rhythms such as the cardiac and the respiratory cycles can affect both the intrinsic dynamics of the brain and awareness. The cardiac muscle cyclically contracts and ejects blood during the systole and then relaxes during the diastole to allow refilling of the chambers. During the systole, baroreceptors (BRs) in the aortic arch and carotid sinus detect increases in blood pressure and signal the brainstem to regulate the heart rate via the baroreflex. BR signals are then further relayed to higher cortical areas, which in turn modulate how the brain processes different types of external stimuli. In effect, somatosensory (Al et al., 2020; Grund et al. 2022; Motyka et al. 2019), auditory (Schulz et al. 2009) and visual (Birren et al. 1963; Pramme et al. 2014; Sandman et al. 1977) stimuli are better perceived during the diastole when BRs are less active. Moreover, somatosensory evoked potentials have higher amplitude for stimuli presented during the diastole than the systole (Al et al., 2020). According to the BR hypothesis (Lacey and Lacey 1958), cortical excitability decreases with BR stimulation affecting cortical gain. Gain refers to the amount of input required to produce a response: when gain is high, relevant stimuli are amplified and irrelevant ones reduced to optimize sensory processing. Conversely, when cortical excitability and thus gain are low, relevant and irrelevant stimuli are less differentiated and sensory processing is less effective (Eldar et al. 2013). Perceptual efficiency fluctuates across the cardiac cycle and it is reduced during the systole when BR are most active and increases during the diastole when BR activity is lower (Skora et al. 2022).

Respiration is another fundamental bodily rhythm whose primary function is the exchange of oxygen for carbon dioxide. During inhalation, the airflow mechanically stimulates the olfactory bulb, leading to the entrainment of cortical rhythms beyond olfactory regions (Herrero et al. 2018; Zelano et al. 2016). This effect is greatly reduced during oral breathing which stimulates the OB to a lesser extent (Zelano et al. 2016). Respiration affects also broad-band MEG resting-state activity (Kluger and Gross 2021), alpha power fluctuates with the respiratory phase and both perceptual sensitivity and its association with alpha power are enhanced during inhalation (Kluger et al. 2021; Kluger and Gross 2021) suggesting that inhalation corresponds to a state of heightened cortical excitability.

Overall, both BR activity and respiratory phase can modulate both cortical excitability and cortical gain and thus the state of the brain. It is important to consider that these factors are not independent: BR activity plays a fundamental role in coupling the cardiac frequency to the respiratory phase to optimize gas exchange through respiratory sinus arrhythmia (RSA). During inhalation, oxygen is more available and therefore, BR activity decreases to accelerate the heart rate. Conversely, during exhalation, BR activity increases and triggers the baroreflex, slowing down the heart rate (Noble and Hochman 2019). Since BR activity increases during exhalation, it might reduce cortical excitability and gain independently of the stimulation of the OB.

We have previously shown that the cardiac and respiratory phases modulate both the cortical regions involved in awareness (frontal cortex for low and parietal for high BR activity) and the earliest marker of awareness (Leupin and Britz 2024). In particular, the early sensory component (P1) is modulated by awareness only when BR activity and thus cortical gain is low during the diastole and inhalation. This modulation is abolished when subjects breathe through the mouth which greatly reduces the stimulation of the OB (Leupin and Britz 2025a). These findings indicate that the mode of breathing differently affects early sensory components which are more sensitive to cortical excitability (Iemi et al. 2019). Overall, both the cardiac and respiratory phases and the mode of breathing affected the evoked potentials related to awareness (Leupin and Britz 2024, 2025a, 2025b) indicating that the brain aligns its mode of processing with the physiological state of the body. Given that the momentary state of the brain preceding the presentation of the stimulus similarly varies for aware vs. unaware (Britz et al. 2014) and multi-stable stimuli (Britz et al. 2009, 2011), it might be equally affected by trial-to-trial variations in physiological signals from the body.

In the present study we analyze the data from the subjects in Leupin and Britz (2024) to investigate whether differences in the pre-stimulus microstates preceding aware and unaware trials interact with the cardiac and respiratory phase similarly to the ERPs. We used an orientation discrimination task where subjects had to discriminate between left and right oriented Gabor gratings embedded in random dot noise and to report whether they perceived the stimulus. We compared the same physical stimulus when it was correctly discriminated with and without awareness (Eklund and Wiens 2018; Britz et al. 2014) to control for possible confounds between awareness and performance (Lau and Passingham 2006). We first expect to replicate the findings of Britz et al. (2014) and identify two microstate templates which dissociate the correct aware and correct unaware condition. If the cardiac and respiratory cycles interact with the momentary state of the brain, we expect to find one or multiple microstates to be more present as a function of awareness (aware/unaware) depending on the cardiac (systole/diastole) and respiratory (inhalation/exhalation) phase. If this effect is modulated by BR activity, then the microstate preceding the unaware state should be more prominent during the systole and exhalation when BR activity is stronger.

## Methods

### Participants

Forty healthy subjects (26 female, age: 24.6 ± 5 years, range 18–42) were recruited for the EEG study. All participants reported no history of neurological, psychiatric, cardiological and respiratory disorders and were right-handed (Oldfield 1971). The discrimination threshold could not be determined for six subjects, and data from five subject were excluded due to compromised data quality of the ECG (three subjects) and of the EEG (two subjects). Data from 29 subjects (17 female, age 24.42 ± 4.9 years, range 18–42) were retained for analyses. The Ethics Committee of the University of Fribourg approved the informed written consent provided by the participants and the study was conducted in accordance with the Declaration of Helsinki. Subjects gave written informed consent and were rewarded either with monetary compensation (20 CHF/hour) or course credits.

### Stimuli and Procedure

**Fig. 1:**
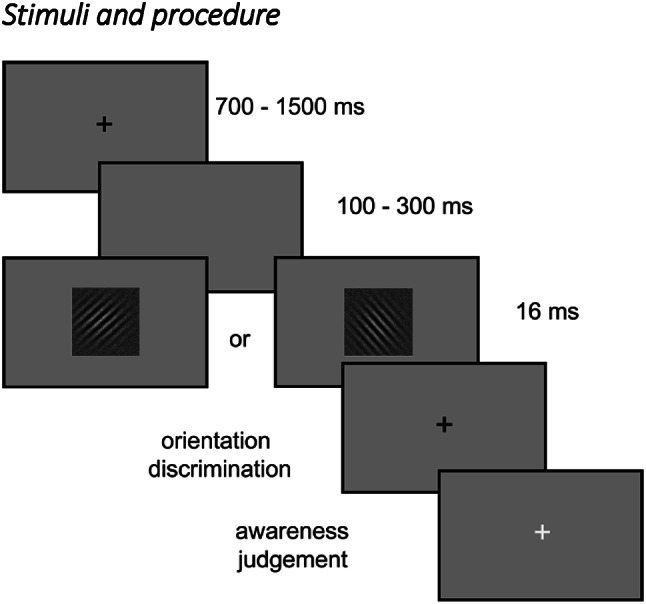
Experimental procedure. A Gabor grating either oriented to the left or to the right was presented for 16 ms. Subjects first had to indicate the orientation of the stimulus (accuracy measurement) and then whether they saw the stimulus or whether they guessed (awareness measurement)

Figure 1 depicts the stimuli and experimental procedure. The stimuli were Gabor gratings embedded in grayscale random dot noise that were either oriented to the left (135°) or the right (45°). They subtended a visual angle of 5° with 3 cpd of visual angle. Psychopy3 was used to both produce and display stimuli on a grey background on a ViewPixx Screen (1920 × 1080 pixel resolution, 120 Hz). Participants first completed a threshold determination task and then performed the main EEG experiment. Subjects were positioned on a chin-rest 70 cm away from the screen in a dimly lit room and were directed to breathe exclusively through their nose, with a small strip of surgical tape lightly placed over their lips to prevent breathing through the mouth.

At the beginning of each trial, a white fixation cross appeared for a duration ranging between 700 and 1500 ms, followed by a blank screen (100–300 ms) and then the target stimulus was briefly presented for 16 ms. Participants responded by indicating the orientation of the grating using a keyboard (“F” key with their left index for a leftward / “J” key with their right index finger for a rightward orientation). Afterward, they reported whether they perceived the stimulus (“J” if they saw it, “F” if they did not). These responses provided measures of both objective accuracy and subjective awareness of the stimulus.

Before the EEG experiment, participants completed a threshold determination procedure designed to account for both task performance and subjective awareness. To disentangle these factors, we ensured that accuracy remained consistently high (> 75%) while maintaining the same proportion of correct aware and correct unaware trials. The perceptual threshold was titrated by linearly varying the Michelson contrast of the random dot noise in 20 steps, while keeping Gabor grating strength and random dot mask opacity constant (Samaha et al. 2016). Stimuli were presented in a pseudo-randomized order across 5 blocks (10 repetitions for each stimulus) for a total of 400 hundred trials.

In the EEG task, we selected the contrast levels that yielded the correct identification in over 75% of the trials while balancing identification rates between aware and unaware conditions for both the left- and right-facing stimuli. The EEG task included a total of 960 stimuli, distributed across 12 blocks, each block containing 80 trials. We ensured the threshold stability by readjusting the noise contrast throughout the task if necessary.

### Electrophysiological Recordings Data Processing

The EEG was recorded continuously from 128 active Ag/AgCl electrodes (BioSemi^®^) referenced online to the CMS-DRL ground loop. The cardiac (ECG) and respiratory signals were simultaneously recorded by ECG electrodes positioned on the right clavicle and lower left rib and by a breathing belt (SleepSense^®^) placed on the lower abdomen as external bipolar channels with the EEG, and all data were digitized at 1024 Hz/16 bit.

### Preprocessing of Cardiac and Respiratory Signals

The Python Neurokit2 toolbox (Makowski et al. 2021) was used to preprocess the cardiac and respiratory signals. Markers indicating the start of systole and diastole were determined by detecting respectively the R-peak and the end of the T-wave in the ECG signal. Similarly, the inhalation peak and exhalation trough were detected to mark the beginning of the inhalation and exhalation phases in the respiratory signal. Trials were then categorized according to the cardiac and respiratory phases in which they fell. Because the diastole can be almost twice as long as the systole, we equalized the number of trials across the cardiac cycle by including only the stimuli falling within the interval at the end of diastole corresponding to the duration of systole in that particular cardiac cycle (Al et al., 2020; Leupin and Britz 2024). Respiratory cycles deviating by more than 2.5 standard deviations faster or 1.5 standard deviations slower than the mean were excluded from further analysis. We only retained correct trials with (aware) and without awareness (unaware) for further analysis.

### EEG Preprocessing and Microstate Analysis

EEG preprocessing was performed using the MNE-python toolbox version 0.24.0.1 (Gramfort et al. 2013). Because of the CMS-DRL ground loop, Biosemi data are physically unreferenced; to convert the raw signals into meaningful voltages, we applied the common average reference as the first preprocessing step. Afterwards, the signal was band-pass filtered between 0.5 and 40 Hz using a FIR filter with a transition window of 10 Hz and down sampled to 256 Hz. We applied Independent Component Analysis (ICA) to remove ocular and myogenic artifacts and discarded the trials with ocular artifacts occurring within 300 ms before or after stimulus presentation. The respective ICs were removed to further correct for residual ocular and myogenic artifacts. The data was then segmented into epochs spanning from − 200 ms to 1000 ms relative to stimulus onset. Additionally artifact rejection and channel interpolation were performed using the Autoreject procedure (Jas et al. 2017) implemented in MNE.

Microstate analyses were conducted using the Pycrostates toolbox version 0.2.0 (Férat et al. 2022). Microstates refer to global patterns of scalp topography that remain stable for brief periods of ~ 60–120 ms before transitioning to another stable topography (Lehmann et al. 1998). In periods of stable topography, only the strength but not the configuration of the scalp field varies. Field strength is reflected by the global field power (GFP), calculated as the spatial standard deviation of the potential field (Lehmann and Skrandies 1980). Because only the strength varies between two troughs of the GFP, the local maxima of the GFP are the best representative of a given microstate in terms of maximal signal-to-noise ratio. For our analysis, we considered only the microstate immediately preceding a stimulus as it crucially contributes to its outcome (Britz et al. 2009, 2011, 2014; Kondakor et al. 1997; Kondákor et al. 1995; Lehmann et al. 1998; Mohr et al. 2005).

The microstates analysis were performed in four steps. Given that microstates last roughly between 50 and 100 ms and that local peaks of the GFP are the best representative of a given microstate in terms of signal to noise ratio, we first, we extracted the map at the GFP maximum closest to stimulus onset in the 50 ms time-window before stimulus onset for each epoch in all subjects. Second, we submitted those maps to k-means cluster analysis (Pascual-Marqui et al. 1995) over the whole sample to identify the topographic maps which best characterized the data. We did not assume a priori the number of clusters but instead used data-driven metrics to evaluate the best fit. We performed cluster analysis ranging from 2 to 20 clusters and determined the number of clusters that best explained the data using and aggregate of the Silhouette (Rousseeuw 1987), (Caliński and Harabasz 1974), (Dunn 1974), and (Davies and Bouldin 1979) metrics implemented in the pycrostates toolbox to evaluate the quality and distinctiveness of the clusters. These scores evaluate different aspects of the distance between two data-points belonging to two different clusters (inter-cluster distance) and the same cluster (intra-cluster distance). The Silhouette describes the consistency of each cluster considering both the inter- and intra–cluster distance. The Calinski-Harabsz measures the ratio of the inter and intra cluster dispersion. The Dunn evaluates the goodness of separation of the clusters and finally the Davies- Boulding represent how similar (ratio of intra to inter-cluster distance) a cluster is to its most similar cluster. Third, after determining the optimal number of clusters, each template map was spatially correlated to the topography of each trial and the best match retained. For each subject and condition (awareness, cardiac and respiratory phase) we computed the global explained variance (GEV) and time coverage metrics. The GEV indicates how strongly each template represents the data, and it is calculated as the sum of the explained variance weighted by the GFP. The time coverage measures the percentage of epochs in which each microstate template was present. Fourth, we performed 2 × 2 repeated measure ANOVAs for each map, investigating the effects of awareness (aware/unaware) and cardiac phase (systole/diastole), and of awareness (aware/unaware) and respiratory phase (inhalation/exhalation) separately for the GEV and time-coverage. All statistical analyses were performed using R Statistical Software (v 4.3.2; R Core Team 2023). Images were produced using the Seaborn (Waskom 2021) package (version 0.11.2).

## Results

Subjects responded correctly in 85.3% (*SD* = 6.4%) of trials with a mean ratio of correct aware/unaware responses of 50.4%/49.6% (*SD* = 11.7%). Only correct trials were submitted to microstates analysis.

**Fig. 2:**
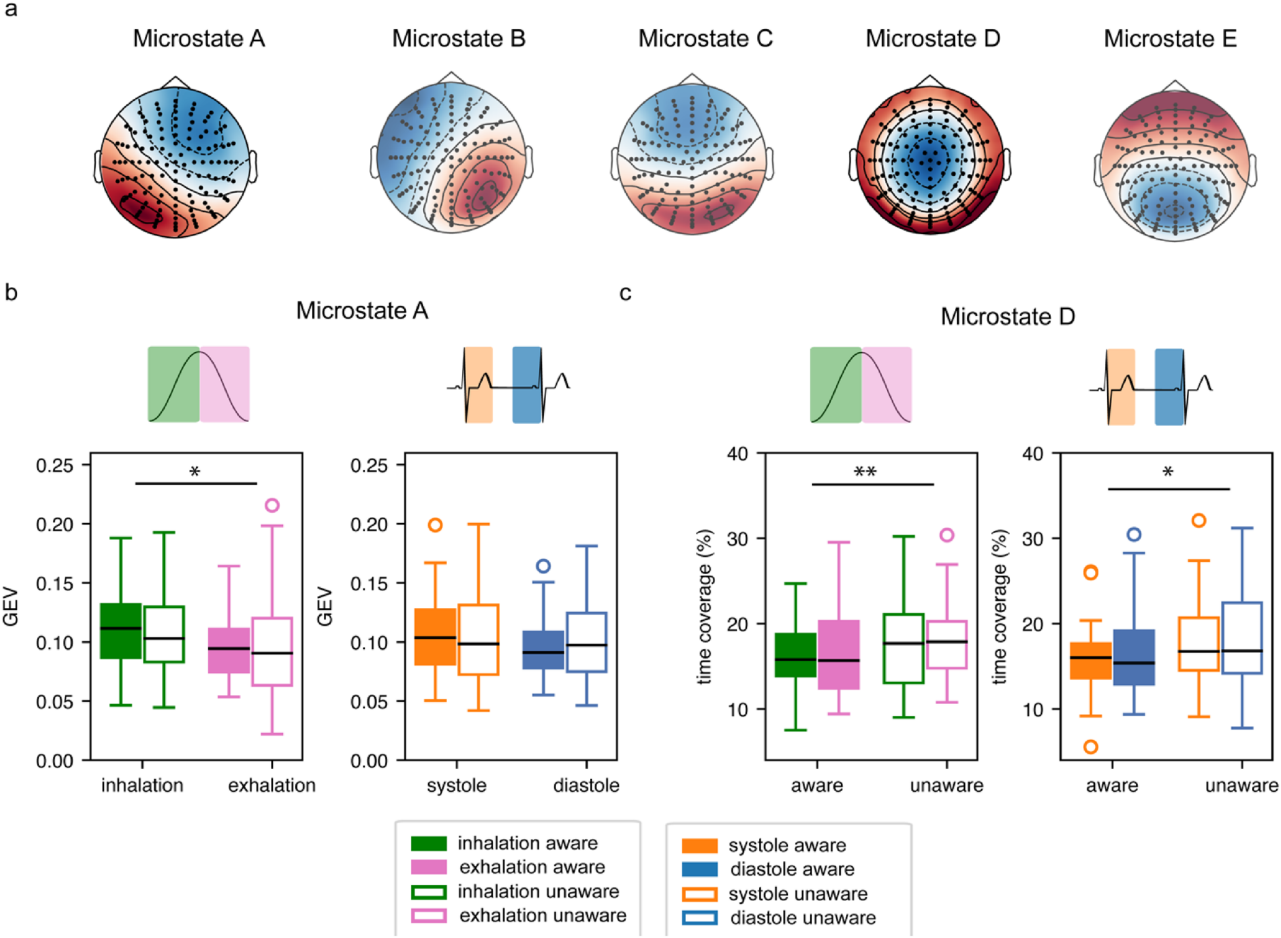
Pre-stimulus EEG microstates results. **a**) The five microstate topographic map obtained from the clustering relabelled according to the canonical maps. The **b**) global explained variance (GEV) of Microstate A and **c**) time coverage of Microstate D expressed as a function of the respiratory (inhalation in pink/exhalation in green) or the cardiac phase (systole in orange/diastole in blue) and of awareness (aware filled/unaware empty). The boxplots show the median (mid-line), the 25–75% percentile (box), the 1.5 interquartile range (whiskers) and the outliers (dots). * *P* <.05, ** *P* <.01

After artifact rejection, an average of 254 trials were retained in the correct aware condition (128/127 in the systole/diastole and 115/139 in inhalation/exhalation) and 236 in the correct unaware condition (123/125 for systole/diastole and 113/134 for inhalation/exhalation). Out of the range of 2 to 20 clusters produced from the k-means cluster analysis, the evaluation metrics identified a five-cluster solution as the best fit (Fig. 2a), which explained 61.2% of the Global Variance. The obtained clusters were then relabelled according to the canonical topographies from A to E (Tarailis et al. 2023). Subsequently, each trial was assigned the template map displaying the highest spatial correlation to determine the GEV and the time coverage of each map in each condition.

Figure 2 summarizes the results of the ANOVAs performed on the GEV of Microstate A (Fig. 2b) and the time coverage of Microstate D (Fig. 2c), as a function of the bodily phase (systole/diastole and inhalation/exhalation) and awareness. Microstate A had significantly higher GEV (F(1,84) = 5.2, *p* =.024, *n*^*2*^*p* = 0.06) during inhalation (*M* = 0.11, *SD* = 0.04) compared to exhalation (*M* = 0.01, *SD* = 0.04). There were no significant main effects of awareness (F < 1), nor any significant interaction effects between respiratory phase and awareness (F < 1). Microstate D was significantly more prevalent, i.e. it had a higher time coverage in the unaware (*M* = 18%, *SD* = 5%) than in the aware (*M* = 16%, *SD* = 5%) condition for the cardiac ANOVA (F(1,84) = 6.3, *p* =.014, *n*^*2*^*p* = 0.07). We found the same main effect of awareness (aware *M* = 16%, *SD* = 4%, unaware *M* = 18%, *SD* = 5%) in the respiratory ANOVA *F*(1,84) = 7.9, *p* =.006, *n*^*2*^*p* = 0.09. However, no interaction effect was observed between awareness and the cardiac (F < 1) or the respiratory phase (F < 1). Similarly, there was no main effect either of cardiac (F < 1) or of respiratory phase (F < 1). None of the ANOVAs for the remaining maps yielded significant results (all *p* >.05).

## Discussion

In the present study we address whether spontaneous fluctuations in brain activity as indexed by EEG microstates and cyclic variations of bodily signals exert a joint or independent influence on fluctuations in subjective awareness. We used an orientation discrimination paradigm where the same physical stimulus was correctly identified with or without awareness while maintaining accuracy near ceiling.

We show that pre-stimulus microstates differences predict awareness irrespective of cardiac and respiratory phase and that they differed with the respiratory but not the cardiac phase. Moreover, these effects did not interact, i.e. pre-stimulus microstates predicted differences in awareness independently of the cardiac and the respiratory phase.

First, we observed that Microstate D was more present in the pre-stimulus period preceding the correct unaware compared to the correct aware condition irrespective of the cardiac or respiratory phase. Several studies identified the intracranial sources of this microstate within fronto-parietal regions associated with the dorsal attentional network (DAN) (Bréchet et al. 2019; Britz et al. 2010; Custo et al. 2017), which reflects attentional processes. Typically, microstate D is more frequently observed under conditions of high cognitive load that demand significant attentional resources compared to rest or easier tasks (Bréchet et al. 2019; Kim et al. 2021). However, Milz and collegues (2016) observed longer durations of microstate D during rest compared to goal-directed task and suggested that it represents reflexive aspects of attention which are not optimal during a focused task. In line with these findings, Britz et al. (2010) reported microstate D to be *anti*correlated with BOLD signal in the DAN indicating that the presence of this microstate corresponds with a deactivation of this network. Pre-stimulus deactivation of the DAN is associated with lapses in attention preceding errors (Weissman et al. 2006) which explains why we observe that Microstate D is more present before trials where the subjects fail to consciously perceive the stimulus despite providing the correct response. Contrary to Britz et al. (2014), who identified two distinct microstate topographies that differentiated the correct aware and correct unaware condition in a backward masking paradigm, our paradigm revealed only one microstate topography which covered more time in the correct unaware compared to the correct aware condition. This discrepancy could be due to differences in the nature of the task: while in the present study, we used a single stimulus (Gabor patch embedded in random dot-noise), Britz et al. (2014) used backward masking to elicit subjective difference in awareness. Backward masking relies on the precise timing of a second masking stimulus to disrupt re-entrant recurrent processing of the primary stimulus coming from higher visual cortices to lower level regions (Fahrenfort et al. 2007). Thus, the pre-stimulus processes which contribute to the perceptual outcome of a single stimulus appear to differ from those interacting with the stimulus and its mask.

Next, we find that Microstate A explained more variance during inhalation compared to exhalation. A recent review (Tarailis et al. 2023) pointed out a consistent connection between microstate A and the degree of subjective arousal, as it occurs more often in states of high than low arousal (Antonova et al. 2022; Ke et al. 2021). Since microstate A explains more variance during inhalation than exhalation it might reflect higher arousal during inhalation. This is in line with findings of coupling between alpha power and inhalation (Kluger et al. 2021) which result in higher detection rates in this phase. Despite these findings, respiratory phase and awareness did not interact over the global variance explained by microstate A, implying that global pre stimulus processes affecting subjective awareness are independent of respiratory-induced cortical changes.

Neither the cardiac phase nor its interaction with awareness was significantly associated with changes in the microstate topography within the pre-stimulus period. This finding is unexpected considering the well-established literature on baroreceptor activity modulating the perceptual outcome of stimuli through changes in cortical excitability and gain (Skora et al. 2022). Baroreceptor-modulated gain control explains sensory processing differences in the evoked response across both the cardiac and respiratory phases (Leupin and Britz 2024). This effect is abolished for the respiratory phase and delayed for the cardiac phase when the same participants breathe through their mouth suggesting that OB stimulation might play a role in sustaining the cortical gain in sensory cortices (Leupin and Britz 2025a). While these evoked differences are related to gain control, this is not reflected in the pre-stimulus global state of the brain as indexed by EEG microstates: we do not observe any interaction between the cardiac and respiratory phases and awareness. Given that each microstate has its independent physiological significance, we separately assessed their influence on awareness in conjunction with bodily phase. Because of that we did not control for multiple comparisons, which could be considered a limitation of the study.

Post-stimulus ERPs and pre-stimulus microstates provide complementary insights on the process underlying conscious awareness. Future research should address the link between physiological phase and pre-stimulus microstates performing microstate analysis at rest over longer periods of time which would allow to investigate the duration and transition probabilities of each map. These dynamical measures might help clarify how the cardiac and respiratory phase affect the global state of the brain.

Taken together, we show that pre-stimulus microstates contribute to determine the perceptual outcome of the stimulus. However, the physiological state of the body seems to affect the momentary state of the brain only as a function of the respiratory cycle without interacting with the perceptual fate of the stimulus.

## Data Availability

The code developed during the current study is available in the *leupin_britz_micro* (Leupin, 2025) repository on Zenodo https://zenodo.org/records/15173349. The consent forms signed by participants do not allow us to give free access to data but require us to check that data are shared with members of the scientific community. Therefore, data are not shared publicly but can be made available upon request to researchers. Please contact the corresponding author Juliane Britz ([juliane.britz@unifr.ch](mailto: juliane.britz@unifr.ch)).
